# The lncRNA MEG3 downregulation leads to osteoarthritis progression via miR-16/SMAD7 axis

**DOI:** 10.1186/s13578-017-0195-x

**Published:** 2017-12-13

**Authors:** Jin Xu, Yaozeng Xu

**Affiliations:** 1 Department of Orthopedics, The First Affiliated Hospital of Soochow University, No. 899 Ping Hai Road, Gusu District, Suzhou, 215031 China; 2 Department of Orthopedics, Baoshan District Shanghai Integrated Traditional Chinese and Western Medicine Hospital, Shanghai, 201999 China

**Keywords:** lncRNAs MEG3, miR-16, SMAD7, Osteoarthritis

## Abstract

**Background:**

Osteoarthritis (OA) is a chronic joint disease and there is no a definitive cure at present. Long non-coding RNAs (lncRNAs) have been confirmed to play important roles in the development of OA. However, the underlying mechanism of lncRNA maternally expressed gene 3 (MEG3) in OA has not been well elucidated.

**Methods:**

The rat OA model and interleukin-1β (IL-1β)-induced rat chondrocytes were constructed. The expression pattern of lncRNA MEG3 and miR-16 was detected by RT-qPCR assay in cartilage tissues of rat OA model. The effect of MEG3 and miR-16 on IL-1β-induced chondrocytes was evaluated on the basis of cell viability and apoptosis. Then, the interaction among MEG3, miR-16 SMAD7 was explored by dual-luciferase reporter assay and RIP assay.

**Results:**

It is found that lncRNA MEG3 was down-regulated and miR-16 was up-regulated in rat OA cartilage tissues. MEG3 knockdown promoted proliferation and inhibited apoptosis, while miR-16 knockdown suppressed proliferation and promoted apoptosis in IL-1β-induced rat chondrocytes. Moreover, MEG3 was involved in miR-16 pathway and MEG3 suppressed miR-16 expression. Additionally, SMAD7 was a target gene of miR-16 and miR-16 suppressed SMAD7 expression in IL-1β-induced chondrocytes. Moreover, the expression of SMAD7 induced by MEG3 or si-MEG3 was markedly reversed by the introduction of miR-16 or anti-miR-16. Furthermore, MEG3 exerted its anti-proliferation and pro-apoptosis by regulating miR-16 and SMAD7.

**Conclusion:**

MEG3 was down-regulated and miR-16 was up-regulated in cartilage tissues of rat OA model. MEG3 knockdown might lead to the progression of OA through miR-16/SMAD7 axis.

## Background

Osteoarthritis (OA) is a common chronic joint disease, mainly characterized by the cartilage loss, the new bone formation of joint surface as well as osteophyte formation [1]. There is a high incidence of OA at aged 65 and older, which is becoming a major public health problem [2]. Recent studies have verified that OA is highly hereditary disease and closely associated with inflammatory response [1]. Although the early diagnosis of OA is more accurate through the X-ray and new biomarkers, the OA causes remain unknown and a definitive cure is still not available [3]. Therefore, it is essential for its treatment to explore the molecular mechanism of OA.

Long non-coding RNAs (lncRNAs), a series of noncoding endogenous RNAs comprising a sequence larger than 200 nucleotides (nt), have been confirmed to play important roles in the development of inflammation-related diseases [4]. Cui et al. [5] verified that lnc-IL7R was able to suppress the LPS-induced inflammatory response. Increasing evidences indicated that a series of lncRNAs had vital functions in the progression of OA [6], for example, HOTAIR and PCGEM1 Upregulation of HOTAIR contributes to IL-1β-induced MMP overexpression and chondrocytes apoptosis in temporomandibular joint osteoarthritis [7]. PCGEM1 stimulates proliferation of osteoarthritic synoviocytes via targeting miR-770 [8]. Maternally expressed gene 3 (MEG3), a maternally expressed lncRNA, had closed relationship with flammation-related diseases, including OA [9]. Recently, Su et al. [9] discovered that MEG3 was downregulated in OA tissues and MEG3 might be involved in OA development and progression by inhibiting VEGF expression levels. However, the underlying molecular mechanism of MEG3 in OA has not been well illustrated.

Similar with lncRNAs, microRNAs (miRNAs), a type of small non-coding RNAs with 18–22 nt in length, also play important roles in a series of inflammation-related diseases [4]. Recent studies revealed that miR-19b, miR-30a, miR-301a promoted the progression of periodontitis [4] and miR-146a, miR-98 were upregulated to contribute to the progression of OA [10]. Increasing evidence revealed that miR-16 might be essential for cell apoptosis by hindering Bcl-2 expression in liver fibrosis [11]. Li et al. [12] also confirmed that miR-16-5p contributed to the development and progression of OA by regulating SMAD3 expression.

The competing endogenous RNA (ceRNA) hypothesis suggested that lncRNAs functioned as a ceRNA of miRNAs to play important roles, for example, PCGEM1 acted as a ceRNA of miR-770 in OA [8]. Li et al. [13] discovered that MEG3 repressed the expression of miR-125a-5p in immune thrombocytopenic purpura. Nevertheless, the relationship between MEG3 and miR-16 and their functions in OA remained largely unknown. In this study, it is found that MEG3 was down-regulated and miR-16 was up-regulated in cartilage tissues of rat OA model. Furthermore, our results suggested that MEG3 might repress the progression of OA through miR-16/SMAD7 axis.

## Methods

### Experimental animals and OA model

Male Sprague–Dawley rats (200–250 g) were obtained from Henan Research Center of Laboratory Animal (Zhengzhou, China). The rats were anesthetized by 30 mg/kg pentobarbital sodium, and destabilization of the medial meniscus (DMM) was performed as previously described [14]. Briefly, in DMM group (n = 10), after the right knee joint was exposed by a medial capsular incision, the extensor muscles were gentle moved and the medial meniscus was transected, then the medial capsular incision and the skin were seamed. In sham group (n = 10), the joint capsule was opened but the medial meniscotibial ligament was left intact. After 4 weeks, the rats were killed and cartilage tissues were harvested under sterile conditions. All animals were treated according to the national guidelines of the care and use of laboratory animals with the approval of the Ethics Committee for Animal Research.

### Cell isolation and cell culture

Chondrocytes were isolated and cultured as previously described [15]. In brief, rat cartilage tissues were cut into small pieces and digested with trypsin (Sigma-Aldrich, St. Louis, MO, USA). After digested into monolayer cells, chondrocytes were seeded into culture plate (Corning, Toledo, NY, USA) in DMEM medium (Gibco, Rockville, MD, USA) with 10% FBS (Gibco), 100 U/ml penicillin (Gibco), 100 mg/ml streptomycin (Gibco) at 37 °C. Adherent chondrocytes at 70–80% confluency were cultured in serum-free medium for 12 h, and then stimulated with 10 ng/ml IL-1β for 2 h to mimick OA chondrocytes. HEK 293T cells were purchased from American Tissue Culture Collection (ATCC, Manassas, VA, USA), which was cultured in MEM medium (Gibco) with 10% FBS.

### Cell transfection

The MEG3 and SMAD7 sequences were synthetized from Sangon Biotech (Shanghai, China) and cloned into a pcDNA3.1 plasmid (Thermo Fisher Scientific, Waltham, MA, USA) to construct MEG3 overexpression vector (MEG3) and SMAD7 overexpression vector (SMAD7). All siRNAs (si-MEG3, si-SMAD7, si-NC), miRNAs mimics (miR-16 mimics, miR-NC), and miRNA inhibitors (anti-miR-16, anti-miR-NC) were also obtained from Sangon Biotech. Plasmids or oligonucleotides were transfected into IL-1β-induced chondrocytes by using the Lipofectamine 3000 transfection reagent (Life Technologies, Carlsbad, CA, USA) according to the protocols of manufacturer.

### RNA extraction and RT-qPCR

Total RNA was isolated from cartilage tissues of rat OA model and treated chondrocytes using GenElute™ Total RNA Purification Kit (Sigma-Aldrich) referring to the instructions of manufacturer. 500 ng total RNA was used to detected the relative MEG3 and miR-16 expression by using QuantiNova SYBR Green PCR kit (Qiagen, Hilden, Germany) on an 7500 fast real-time PCR system (Applied Biosystems, Waltham, MA, USA). GAPDH or U6 was used as internal reference and the 2^−ΔΔCt^ method was used to calculate the expression. For the RT-qPCR analysis, the following primers were used: MEG3: 5′-CTGCCCATCTACACCTCACG-3′ (forward) and 5′-CTCTCCGCCGTCTGCGCTAGGGGCT-3′ (reverse); miR-16: 5′-TAGCAGCACGTAAATATTGGCG-3′ (forward) and 5′-TGCGTGTCGTGGAGTC-3′ (reverse); GAPDH: 5′-TGCACCACCAACTGCTTAGC-3′ (forward) and 5′-GGCATGCACTGTGGTCATGAG-3′ (reverse); U6: 5′-GCTTCGGCAGCACATATACTAAAAT-3′ (forward) and 5′-CGCTTCACGAATTTGCGTGTCAT-3′ (reverse).

### Cell viability assays

Cell viability of treated chondrocytes was assessed by Cell Counting Kit-8 (CCK-8, Sigma-Aldrich) referring to the instructions of manufacturer. Briefly, IL-1β-induced chondrocytes were transfected for 24, 48 and 72 h, then 10 μl of CCK-8 solution was added to each well and incubated for 2 h. Subsequently, the absorbance at 450 nm was measured using a microplate reader (Bio-Rad Laboratories, Hercules, CA, USA).

### Flow cytometry

The apoptosis of treated chondrocytes was determined by flow cytometry assay with Annexin V-FITC Apoptosis Detection Kit (Abcam, Cambridge, UK). The apoptotic rate was analyzed with a flow cytometer (FACSCalibur, Becton–Dickinson, Franklin Lakes, NJ, USA) using CellQuest software.

### Western blot analysis

Cells were completely lysed in 200 l of the lysis buffer (Takara, Dalian, China) and then centrifuged at 8000*g* for 5 min. Proteins were separated by 12% SDS-PAGE, and transferred to PVDF membranes (Millipore, Billerica, MA, USA). The membranes were blocked by 5% skimmed milk in TBS for 2 h. After washed three times by TBS containing 0.1% Tween-20 (TBST), the PVDF membranes were incubated with anti-Bax (Cell Signaing Technology, Danvers, MA, USA), anti-Bcl2 (Cell Signaing Technology), anti-SMAD7 (Cell Signaing Technology), anti-β-actin (Cell Signaing Technology) overnight at 4 °C, respectively. After washed with TBST, the PVDF membranes were incubated with HRP-conjugated secondary antibodies (Cell Signaling Technology). Lastly, the PVDF membranes were exposed to ECL Western Blotting Substrate (Solarbio, Beijing, China) for 5 min and were quantified using VersaDoc 4000MP imaging system (Bio-Rad).

### RNA immunoprecipitation (RIP) assay

RNA immunoprecipitation assay was performed by Imprint RNA immunoprecipitation kit (Sigma-Aldrich) referring to the recommended protocols of manufacturer. Firstly, IL-1β-induced chondrocytes were collected and resuspended in RIP lysis buffer (Solarbio), subsequently centrifuged at 12,000*g* for 5 min. Then, cell lysate was incubated with anti-Argonaute2 (anti-Ago2) or anti-IgG (negative control) overnight at 4 °C, followed by the addition of Protein A magnetic beads to get the immunoprecipitation complex. Total RNA was isolated using GenElute™ Total RNA Purification Kit (Sigma-Aldrich). Lastly, the relative enrichment of MEG3 and miR-16 were determined by RT-qPCR analysis.

### Luciferase reporter assay

The partial squences of MEG3 and 3′-UTR of SMAD7 containing the putative binding sites of miR-16 were synthetized and obtained from Sangon Biotech (Shanghai), then were cloned into the pmirGLO Dual-Luciferase miRNA Target Expression Vectors (Promega, Madison, WI, USA) to construct wild-type reporter vectors MEG3 (WT) and SMAD7 (WT), respectively. The mutant MEG3 sequences and 3′-UTR of SMAD7 sequences containing the putative binding sites of miR-16 were performed by Q5 Site-Directed Mutagenesis Kit (New England Biolabs, Ipswich, MA, USA) and then cloned into pmirGLO vectors respectively, to construct mutant-type reporter vectors MEG3 (MUT) and SMAD7 (MUT). The MEG3 (WT) or MEG3 (MUT) were transfected into HEK 293T cells together with miR-NC, miR-16 mimics, anti-miR-NC or anti-miR-16. Similarly, the SMAD7 (WT) or SMAD7 (MUT) were transfected into HEK 293T cells together with miR-NC or miR-16 mimics. HEK 293T cells were contransfected with SMAD7 (WT) and miR-NC, miR-16 mimics, miR-16 mimics + pcDNA, or miR-16 mimics + MEG3. The relative luciferase activity was analyzed by the Dual-Glo Luciferase Assay System (Promega).

### Statistical analysis

Statistical analyses were preformed by Student’s t-test or one-way ANOVA using software SPSS 15.0 (SPSS Inc., Chicago, IL, USA). All data were presented as the mean ± standard deviation (mean ± SD). A P-value less than 0.05 was considered statistically significant.

## Results

### Over-expression of MEG3 inhibited proliferation and promoted apoptosis in IL-1β-induced chondrocytes

**Fig. 1:**
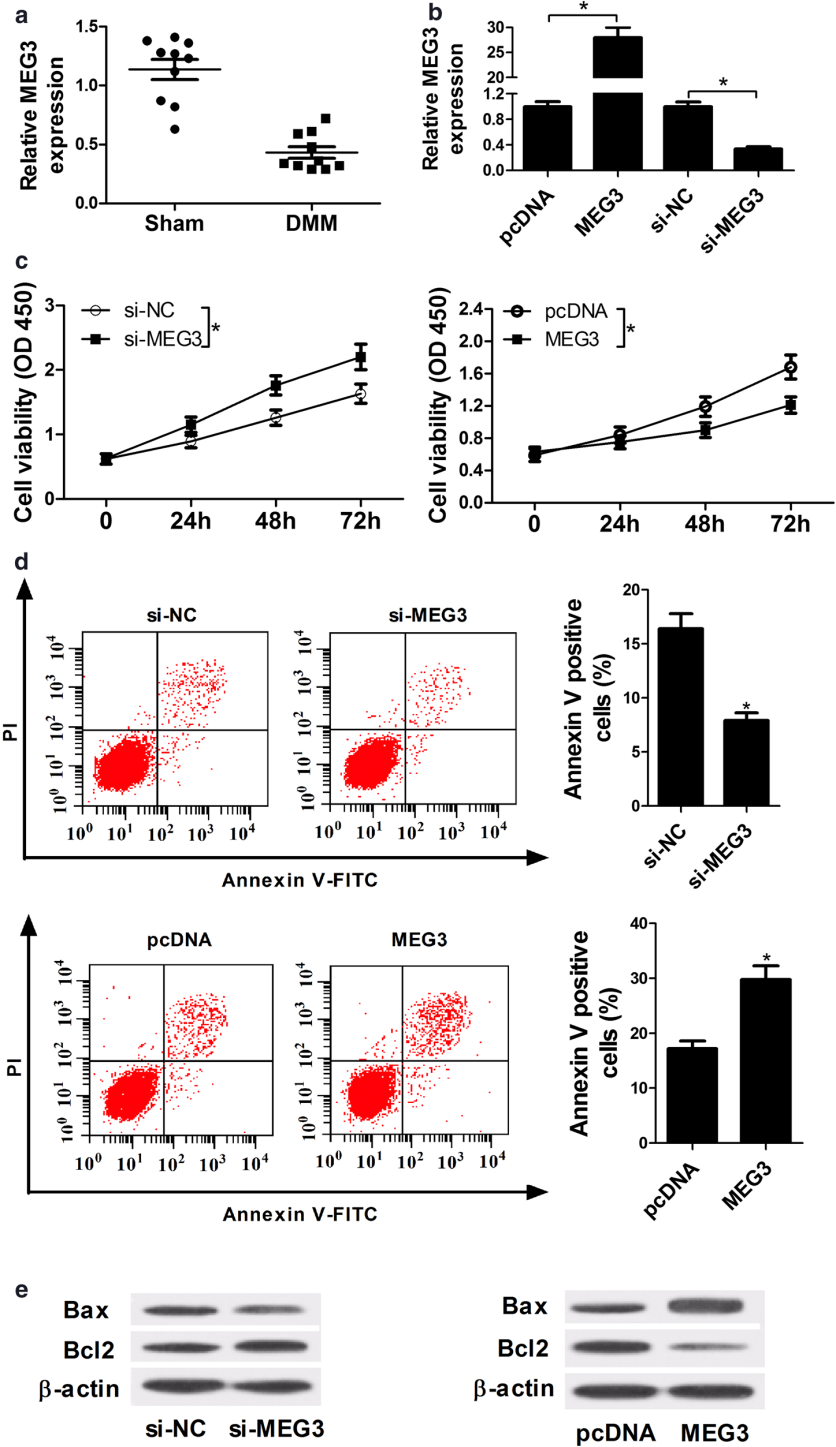
Over-expression of MEG3 inhibited proliferation and promoted apoptosis in IL-1β-induced chondrocytes. **a** The medial meniscus of rats right knee joint was transected to build a rat OA model. MEG3 expression pattern was detected by RT-qPCR in cartilage tissues of rat OA model (n = 10) and sham group cartilage tissues (n = 10). **b**–**e** Chondrocytes were isolated from normal rat cartilage tissues and stimulated with 10 ng/ml IL-1β for 2 h, then transfected with si-NC, si-MEG3, pcDNA-NC or MEG3. **b** After transfection for 48 h, MEG3 expression was detected in IL-1β-induced chondrocytes by RT-qPCR. **c** Cell viability of IL-1β-induced chondrocytes transfected for 0, 24, 48 and 72 h was determined by Cell Counting Kit-8 (CCK-8) at OD450 nm. **d** Cell apoptosis of IL-1β-induced chondrocytes transfected for 48 h was assessed by flow cytometry using Annexin V-FITC Apoptosis Assay kit. **e** Apoptosis-related proteins Bax and Bcl2 expression were detected by western blot assay in IL-1β-induced chondrocytes transfected for 48 h. *P < 0.05 vs. negative control

To investigate the functions of MEG3 in OA, MEG3 expression pattern was detected by RT-qPCR analysis. From the data, it is found that MEG3 expression was drastically decreased in cartilage tissues of rat OA model (n = 10) compared with sham-group cartilage tissues (n = 10, Fig. 1a). Further, chondrocytes were stimulated with 10 ng/ml IL-1β for 2 h to mimick OA chondrocytes. The effect of MEG3 on proliferation and apoptosis of IL-1β-induced chondrocytes was explored using loss-of-function and gain-of-function experiments by transfected with small interference RNA of MEG3 (si-MEG3) and MEG3 overexpression vector (MEG3). As shown in Fig. 1b, the introduction of MEG3 or si-MEG3 markedly enhanced or inhibited the expression of MEG3 compared with the corresponding negative control (pcDNA or si-NC). In addition, cell viability assay revealed that MEG3 knockdown significantly promoted cell proliferation, while MEG3 up-regulation strikingly suppressed proliferation of IL-1β-induced chondrocytes (Fig. 1c). Moreover, the effect of MEG3 on apoptosis of IL-1β-induced chondrocytes was assessed by flow cytometry and western blot assay. The data showed that IL-1β triggered chondrocytes apoptosis and MEG3 down-regulation significantly inhibited IL-1β-induced chondrocytes apoptosis, while MEG3 up-regulation exhibited opposite effect (Fig. 1d). Consistently, western blot assay showed that MEG3 knockdown led to a suppression of apoptosis and MEG3 overexpression led to a promotion of apoptosis, revealed by the expression change of apoptosis-related proteins Bax and Bcl2 (Fig. 1e). In total, the MEG3 overexpression inhibited proliferation and promoted apoptosis in IL-1β-induced chondrocytes. These results suggested that MEG3 might perform an important function in the progression of OA.

### Over-expression of miR-16 promoted proliferation and inhibited apoptosis in IL-1β-induced chondrocytes

**Fig. 2:**
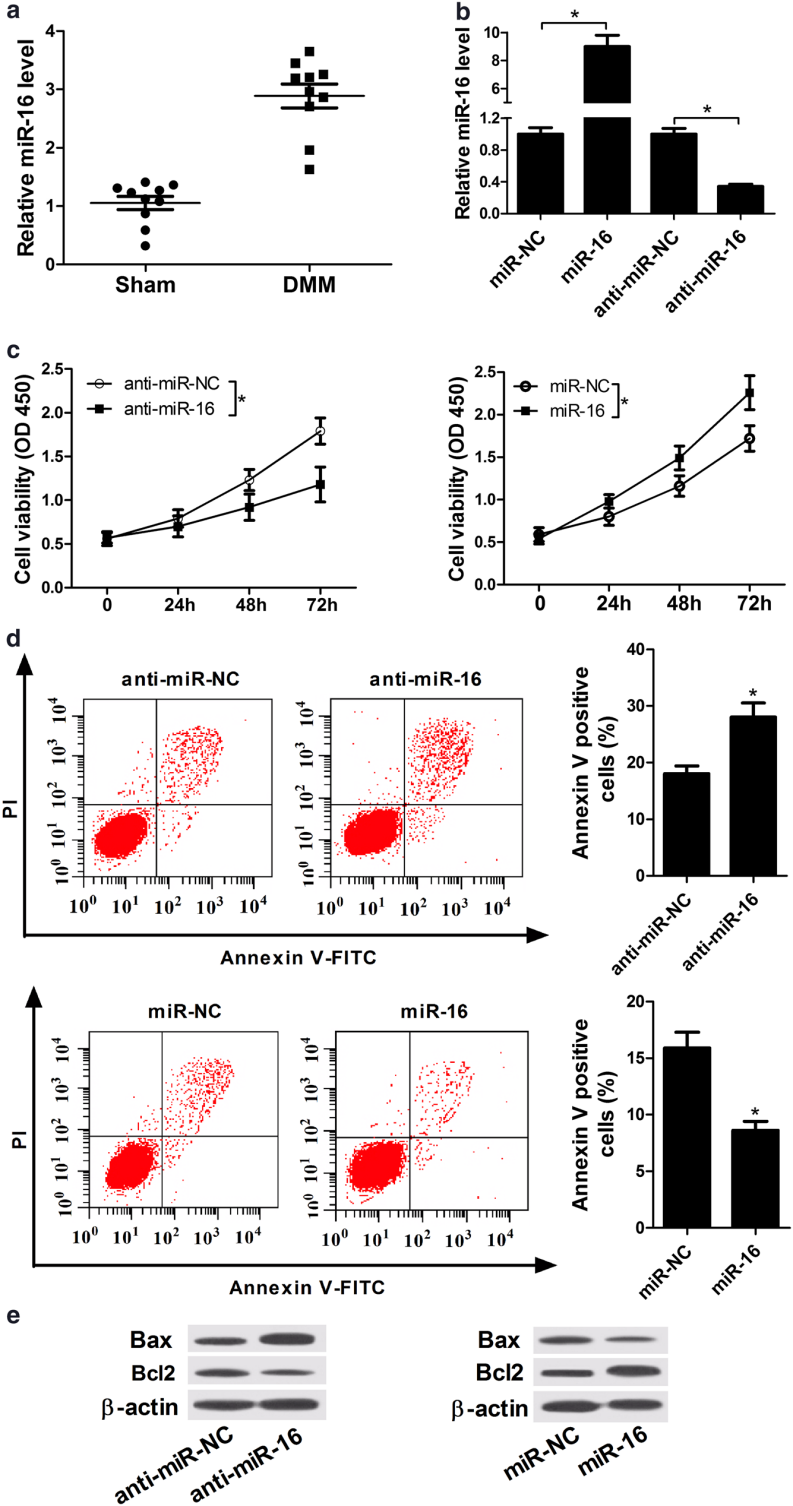
Over-expression of miR-16 promoted proliferation and inhibited apoptosis in IL-1β-induced chondrocytes. **a** The expression of miR-16 was assessed by RT-qPCR in cartilage tissues of rat OA model (n = 10) and sham group cartilage tissues (n = 10). **b**–**e** Chondrocytes were stimulated with 10 ng/ml IL-1β for 2 h, then transfected with miR-NC, miR-16 mimics, anti-miR-NC or anti-miR-16. **b** Relative miR-16 expression was detected in IL-1β-induced chondrocytes transfected for 48 h. **c** Cell viability of IL-1β-induced chondrocytes transfected for 0, 24, 48 and 72 h was determined by Cell Counting Kit-8 (CCK-8) at OD450 nm. **d** Cell apoptosis of IL-1β-induced chondrocytes transfected for 48 h was detected by flow cytometry using Annexin V-FITC Apoptosis Assay kit. **e** Bax and Bcl2 expression were detected in IL-1β-induced chondrocytes after 48 h transfection by western blot analysis. *P < 0.05 vs. negative control

To assessed the effect of miR-16 in OA, miR-16 expression pattern was detected in cartilage tissues of rat OA model. As shown in Fig. 2a, miR-16 expression was evidently increased in cartilage tissues of rat DMM model compared to the sham group. Subsequently, miR-16 mimics and anti-miR-16 were synthesized and then transfected into IL-1β-induced chondrocytes to examine their efficiency. As displayed in Fig. 2b, the expression of miR-16 was markedly increased or decreased by transfecting with miR-16 mimics or anti-miR-16, respectively. Then, miR-16 mimics and anti-miR-16 were used to explore the effect of miR-16 on proliferation and apoptosis of IL-1β-induced chondrocytes. These data revealed that miR-16 depletion markedly inhibited proliferation and promoted apoptosis in IL-1β-induced chondrocytes (Fig. 2c–e). On the other hand, miR-16 overexpression evidently promoted cell proliferation and suppressed cell apoptosis (Fig. 2c–e). In conclusion, the overexpression of miR-16 promoted proliferation and inhibited apoptosis in IL-1β-induced chondrocytes. These results proposed that miR-16 might be closely associated with the development and progression of OA.

### MEG3 was involved in miR-16 pathway

**Fig. 3:**
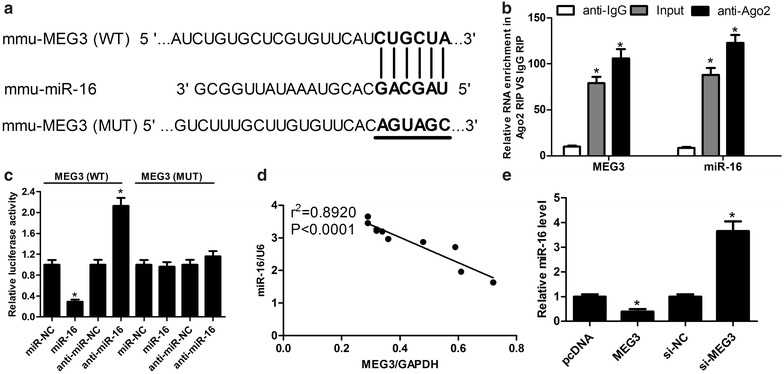
MEG3 was involved in miR-16 pathway. **a** Sequence alignment of miR-16 and the putative binding sites within the wild-type MEG3, and mutation in the MEG3. **b** The interaction between MEG3 and miR-16 was detected by RNA immunoprecipitation (RIP) with Ago2 antibody. **c** The luciferase activity was detected in HEK 293T cells transfected with MEG3 (WT) or MEG3 (MUT) reporter vector together with miR-16 mimics or anti-miR-16. **d** The correlation between MEG3 and miR-16 expression in cartilage tissues of rat OA model by RT-qPCR. GAPDH and U6 were used as internal reference. **e** The relative expression of miR-16 was examined in IL-1β-induced chondrocytes transfected with pcDNA-MEG3, si-MEG3 or control. *P < 0.05 vs. negative control

To further investigate the underlying mechanism of MEG3 in OA, the online software miRcode was used to research for the miRNAs interacted with MEG3. Interestingly, it is found that miR-16 have some complementary bases with the sequences of MEG3, indicating that miR-16 might interact with MEG3 (Fig. 3a). To verify the binding between MEG3 and miR-16, RNA immunoprecipitation (RIP) assay and dual-luciferase reporter assay were performed. Argonaute2 (Ago2) protein is a key components of the RNA induced silencing complex (RISC) and Ago2 antibody may be usefull in capturing mature miRNAs [16]. Therefore, RIP assay was performed using Ago2 antibody to confirm the potentially endogenous interaction between MEG3 and miR-16. The data revealed that MEG3 and miR-16 were largely captured by anti-Ago2 compared with the negative control in IL-1β-induced chondrocytes (Fig. 3b). For dual-luciferase reporter assay, wild-type (WT) and mutant-type (MUT) MEG3 luciferase reporter vectors were constructed and transfected into HEK 293T cells together with miR-NC, miR-16 mimics, anti-miR-NC or anti-miR-16. These results displayed that the luciferase activity of MEG3 (WT) vector was strikingly reduced or enhanced by the transfection with miR-16 mimics or anti-miR-16, respectively. While mutant of putative sites in MEG3 reporter vector had little effect in luciferase activity following miR-16 up-regulation or miR-16 down-regulation (Fig. 3c). In order to further elucidate the interaction between MEG3 and miR-16, the expression of MEG3 and miR-16 were detected in cartilage tissues of rat OA model. As shown in Fig. 3d, MEG3 expression was inversely correlated with miR-16. Moreover, MEG3 over-expression obviously inhibited miR-16 expression, while MEG3 down-regulation markedly promoted the expression of miR-16 (Fig. 3e). Taken together, these results proposed that MEG3 was involved in miR-16 pathway.

### SMAD7 was a target gene of miR-16

**Fig. 4:**
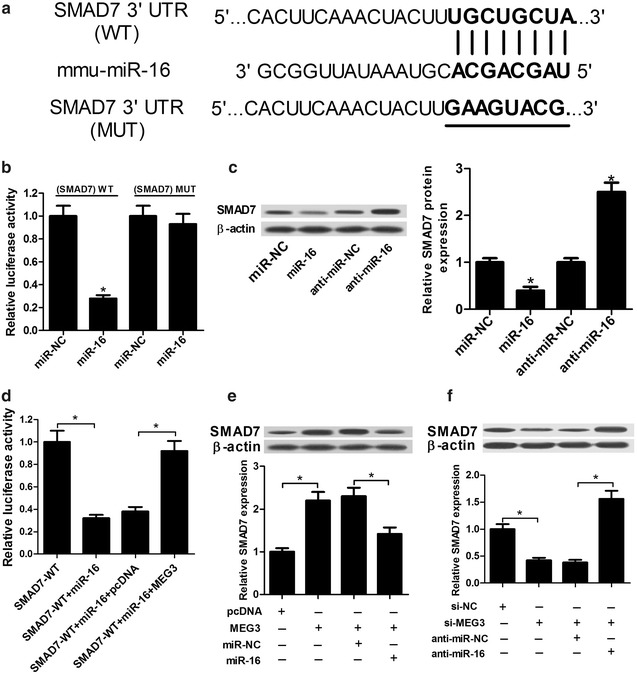
SMAD7 was a direct target of miR-16. **a** Sequence alignment of miR-16 with the putative binding sites within in MEG3 and mutant miR-16 binding sites. **b** Dual-luciferase reporter assays were used to investigate whether SMAD7 could directly interact with miR-16 by the putative binding sites in HEK 293T cells cotransfected with wild-type or mutant-type MEG3 luciferase vectors and miR-NC or miR-16 mimics. **c** SMAD7 expression was detected in IL-1β-induced chondrocytes transfected with miR-NC, miR-16 mimics, anti-miR-NC or anti-miR-16. β-actin was used as internal reference. **d** The effect of MEG3 on luciferase activity in HEK 293T cells transfected with SMAD7 (WT) vector and miR-16 mimics was determined. **e** SMAD7 expression pattern was detected by western blot assay in IL-1β-induced chondrocytes transfected with pcDNA-NC, MEG3, miR-NC or miR-16. **f** SMAD7 expression pattern was detected in IL-1β-induced chondrocytes transfected with si-NC, si-MEG3, anti-miR-NC or anti-miR-16. *P < 0.05 vs. negative control

The online software TargetScan was used to search for the endogenic target gene of miR-16. Intriguingly, it is found that miR-16 have some complementary bases with the 3′-UTR of SMAD7 (Fig. 4a), indicating that SMAD7 might be a target gene of miR-16. Therefore, wild-type SMAD7 luciferase vector (SMAD7-WT) and mutant-type SMAD7 luciferase vector (SMAD7-MUT) were constructed and introduced into HEK 293T cells to verify the interaction between miR-16 and SMAD7. As shown in Fig. 4b, the relative luciferase activity was markedly reduced by the introduction of miR-16 mimics in HEK 293T cells transfected with the SMAD7 (WT) vector, while mutation of the predicated matching sites in the 3′-UTR of SMAD7 had no effect on luciferase activity following miR-16 upregulation. Moreover, western blot assay showed that miR-16 over-expression significantly repressed the expression of SMAD7, while miR-16 down-regulation markedly promoted SMAD7 expression in IL-1β-induced chondrocytes (Fig. 4c).

Based on the above, we further investigated whether MEG3 was involved in miR-16/SMAD7 axis in IL-1β-induced chondrocytes. To validate this assumption, dual-luciferase reporter assay was performed by transfecting SMAD7-WT vector into HEK 293T cells together with miR-NC, miR-16, miR-16 + pcDNA and miR-16 + MEG3. The data revealed that the luciferase activity reduced by miR-16 over-expression in HEK 293T cells transfected with the SMAD7-WTvector, was markedly ameliorated by the introduction of MEG3 (Fig. 4d). On the other hand, MEG3 over-expression markedly promoted SMAD7 expression and MEG3 low-expression strikingly suppressed SMAD7 expression (Fig. 4e, f). Moreover, the expression change of SMAD7 induced by MEG3 or si-MEG3 was markedly reversed by the introduction of miR-16 or anti-miR-16 in IL-1β-induced chondrocytes (Fig. 4e, f). Taken together, our results suggested that SMAD7 was a target gene of miR-16 and MEG3 was involved in miR-16/SMAD7 axis in IL-1β-induced chondrocytes.

### MiR-16 reversed MEG3-mediated anti-proliferation and pro-apoptosis in IL-1β-induced chondrocytes

**Fig. 5:**
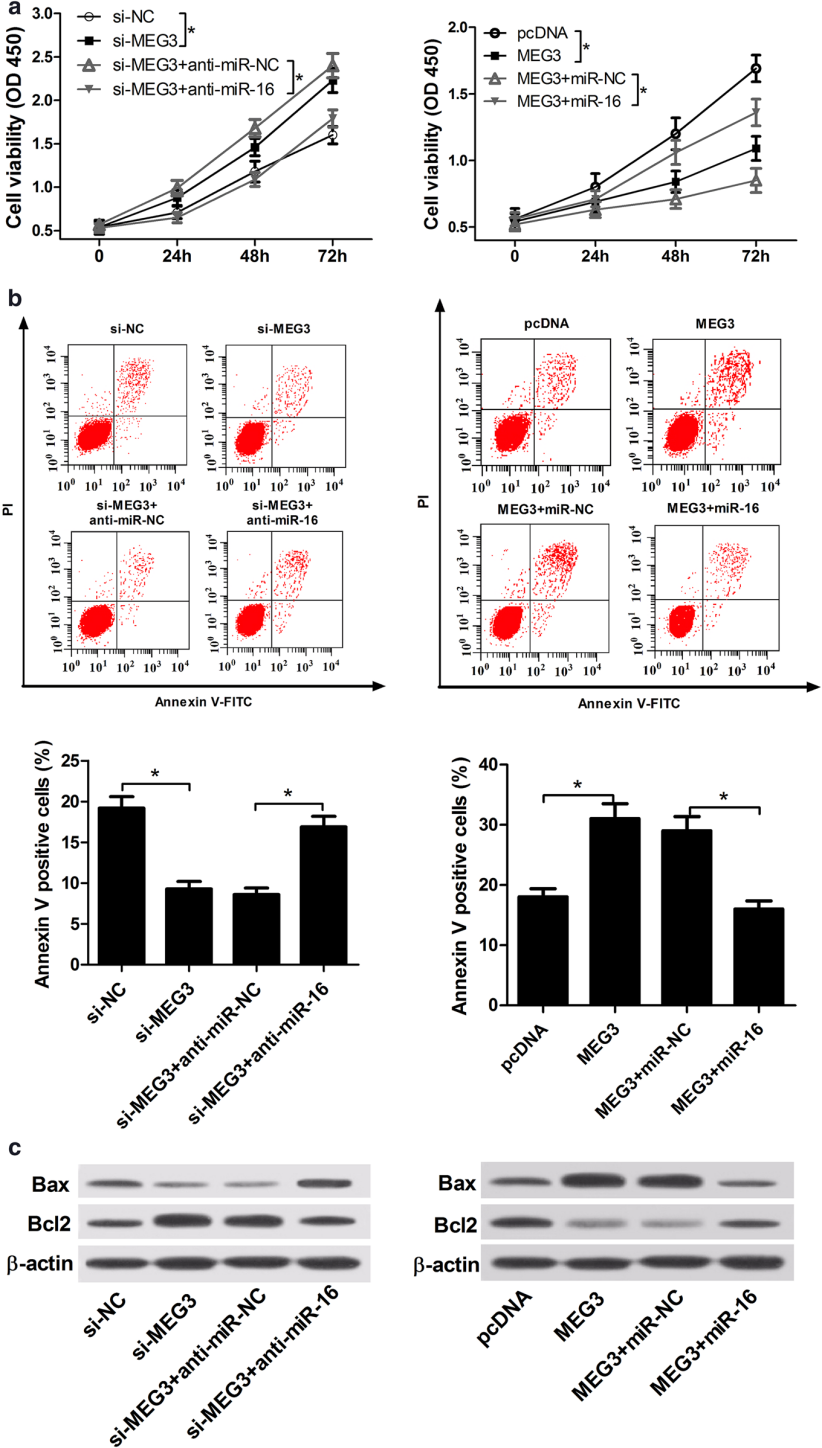
MiR-16 reversed MEG3-mediated anti-proliferation and pro-apoptosis in IL-1β-induced chondrocytes. IL-1β-induced chondrocytes were transfected with si-NC, si-MEG3, si-MEG3 + anti-miR-NC, si-MEG3 + anti-miR-16, pcDNA-NC, MEG3, MEG3 + miR-NC or MEG3 + miR-16 mimics. **a** Cell viability of treated IL-1β-induced chondrocytes was determined at OD450 nm by Cell Counting Kit-8. **b** Cell apoptosis of treated IL-1β-induced chondrocytes was assessed by flow cytometry using Annexin V-FITC Apoptosis Assay kit. **c** Bax and Bcl2 expression were detected in treated IL-1β-induced chondrocytes by western blot analysis. *P < 0.05 vs. negative control

Whether MEG3 exerted its anti-proliferation and pro-apoptosis functions by miR-16 in IL-1β-induced chondrocytes were further explored. As shown in Fig. 5a, cell viability assay revealed that si-MEG3-mediated pro-proliferation effect was significantly reversed by the introduction of anti-miR-16, and MEG3-mediated anti-proliferation effect was evidently alleviated after miR-16 up-regulation. Moreover, si-MEG3-mediated anti-apoptosis effect was markedly abrogated by the introduction of anti-miR-16, and MEG3-mediated pro-apoptosis effect was strikingly reversed by the introduction of miR-16 (Fig. 5b). Consistently, western blot assay also verified the finding of apoptosis showed in Fig. 5b, revealed by the expression change of Bcl2 and Bax (Fig. 5c). In conclusion, these results suggested that miR-16 reversed MEG3-mediated anti-proliferation and pro-apoptosis in IL-1β-induced chondrocytes. In other words, MEG3 might regulate cell proliferation and apoptosis by miR-16 in OA.

### MEG3 exerted its anti-proliferation and pro-apoptosis effect by regulating SMAD7

**Fig. 6:**
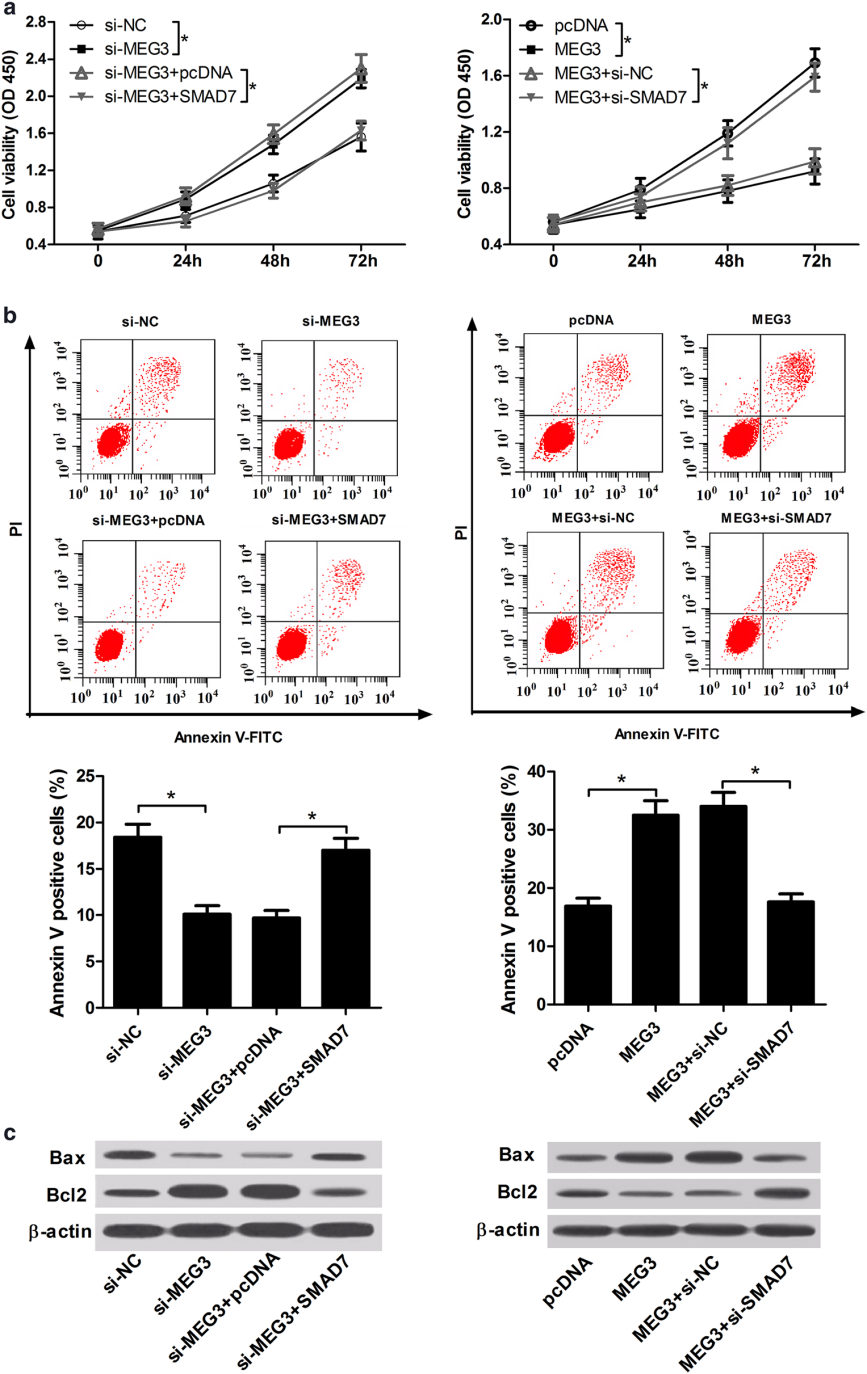
MEG3 exerted its effect on anti-proliferation and pro-apoptosis by regulating SMAD7. IL-1β-induced chondrocytes were transfected with si-NC, si-MEG3, si-MEG3 + pcDNA-NC, si-MEG3 + pcDNA-SMAD7, pcDNA-NC, pcDNA-MEG3, pcDNA-MEG3 + si-NC or pcDNA-MEG3 + si-SMAD7. **a** Cell viability of treated IL-1β-induced chondrocytes was determined. **b** Flow cytometry of cell apoptosis in treated IL-1β-induced chondrocytes. **c** Bax and Bcl2 expression were detected in treated IL-1β-induced chondrocytes. *P < 0.05 vs. negative control

To further investigate the underlying molecular mechanism of MEG3 in OA, cell proliferation and apoptosis were detected in IL-1β-induced chondrocytes transfected with si-MEG3 + PBS, si-MEG3 + SMAD7, MEG3 + PBS, MEG3 + si-SMAD7, or corresponding negative controls. Interestingly, the data showed that si-MEG3-mediated pro-proliferation effect was evidently reversed after SMAD7 up-regulation, while MEG3-mediated anti-proliferation effect was markedly ameliorated by SMAD7 knockdown (Fig. 6a). As shown in Fig. 6b, si-MEG3-mediated anti-apoptosis effect was significantly ameliorated by SMAD7 overexpression, and MEG3-mediated pro-apoptosis was strikingly abrogated by the introduction of si-SMAD7. Furthermore, western blot assay also verified the finding of apoptosis, revealed by the expression change of Bcl2 and Bax (Fig. 6c) in IL-1β-induced chondrocytes. In conclusion, these results suggested that MEG3 might exert its anti-proliferation and pro-apoptosis effect by regulating SMAD7 in OA.

## Discussion

Osteoarthritis (OA) is a common joint disease and has became a major public health problem [1]. Because the underlying mechanism of OA is not fully learned, there is no fundamental therapy [3]. Recently, a series of studies have revealed that lncRNAs play important roles in the development and progression of OA [17]. Some lncRNAs were verified to contribute to OA progression, including HOTAIR [7], GAS5 [18], others could be a potential therapeutic target for OA, including PCGEM1 [8], lncRNA-CIR [19]. LncRNA MEG3 was verified to as a tumor suppressor by targeting p53 in multiple cancers, for example, in meningiomas [20]. Intriguingly, Su et al. [9] discovered that MEG3 was down-regulated and MEG3 might be involved in OA development and progression by the regulation of VEGF levels. In accordance with the findings [9], our data showed that MEG3 expression was drastically decreased in cartilage tissues of rat OA model and MEG3 knockdown significantly promoted proliferation and inhibited apoptosis in IL-1β-induced chondrocytes. The results in this study suggested that MEG3 might perform an important function in OA.

Recently, lots of studies verified the ceRNA hypothesis that lncRNAs functioned as a ceRNA of miRNAs and lncRNAs exerted their function by antagonizing the target miRNAs effects and regulating the expression of miRNAs endogenous targets in a variety of diseases [21]. LncRNA MEG3 also was found to function as a ceRNA of several miRNAs, for instance, miR-125a-5p in immune thrombocytopenic purpura [13], miR-770-5p in Hirschsprung’s disease [22]. Therefore, the online software miRcode was further used to research for the miRNAs interacted between MEG3 in OA. Interestingly, the data showed that miR-16 might interact with MEG3, which was in accordance with a previous report [12]. In present study, MEG3 was involved in miR-16 pathway and MEG3-mediated anti-proliferation and pro-apoptosis effect was abated by miR-16 in IL-1β-induced chondrocytes.

A series of previous studies showed that miR-16 enhanced cell apoptosis by targeting the oncogene Bcl2 in hepatic stellate cells [11], and miR-16 acted as putative tumor suppressor by targeting VEGF-A in multiple myeloma [23]. Intriguingly, Wang et al. [24] found that miR-16 was up-regulation in systemic inflammatory response syndrome (SIRS), which might be useful biomarkers for SIRS diagnoses. In this study, miR-16 expression was significantly increased in cartilage tissues of rat OA model and up-regulated miR-16 elevated cell proliferation and inhibited apoptosis in IL-1β-induced chondrocytes. Increasing evidence showed that miRNAs exerted their function by regulating the expression of endogenous targets [25], then software algorithms was used to search for the target gene of miR-16. Similar with precious studies [26], having shown that caprin-1, cyclin E and HMGA1 were the targets of miR-16 in MCF-7 cell lines, it is verified that SMAD7 was a target gene of miR-16 by dual-luciferase reporter assay and miR-16 markedly repressed SMAD7 expression in IL-1β-induced chondrocytes. Interestingly, previous reports verified that miR-16-5p contributed to the development of OA through targeting SMAD3 [12] and SMAD7 knockdown might contribute to OA development in 6-month old mice [27].

Acted as an intracellular antagonist of TGF-β signaling pathway, SMAD7 plays an important role in many inflammation-related diseases [28]. Montelenoe et al. [29] discovered that SMAD7 down-regulation maintained the chronic production of proinflammatory cytokines to drives the inflammatory respond in inflammatory bowel disease. Lan et al. [30] revealed that SMAD7 played a critical role in anti-inflammation through repressing NF-κB signaling pathway in chronic kidney diseases. As previous reported [29, 30], our results suggested that SMAD7 might play an suppressor role on the development of OA.

In this study, it is found that MEG3 was involved in miR-16/SMAD7 axis in IL-1β-induced chondrocytes. Moreover, MEG3 exerted its anti-proliferation and pro-apoptosis by regulating miR-16 and SMAD7. Taken together, the results in this study suggested that MEG3 might ameliorate the development and progression of OA through regulating miR-16/SMAD7 axis. Similar with our findings, Sun et al. [31] discovered that lncRNA NEAT1 and miR-377-3p had a vital function in non-small cell lung cancer by regulating the target E2F3. Liang et al. [32] also found that lncRNA H19 acted as a ceRNA of miR-138, which antagonized miR-138 effects and regulated the target gene ZEB1 in colorectal cancer.

In conclusion, MEG3 was down-regulated while miR-16 was up-regulated in cartilage tissues of rat OA model, and MEG3 konckdown might lead to promoting proliferation and inhibiting apoptosis in IL-1β-induced rat chondrocytes through miR-16/SMAD7 axis, indicating that MEG3 could be a useful marker and potential therapeutic target in OA.

## Abbreviations

OA: osteoarthritis
lncRNAs: long non-coding RNAs
MEG3: maternally expressed gene 3
IL-1β: interleukin-1β
ceRNA: competing endogenous RNA
anti-Ago2: anti-Argonaute2
RIP: RNA immunoprecipitation
SIRS: systemic inflammatory response syndrome
